# Hexokinases 2 promoted cell motility and distant metastasis by elevating fibronectin through Akt1/p-Akt1 in cervical cancer cells

**DOI:** 10.1186/s12935-021-02312-0

**Published:** 2021-11-10

**Authors:** Qian Chen, Lu Li, Xian Liu, Qian Feng, Yanru Zhang, Pengsheng Zheng, Nan Cui

**Affiliations:** 1 Department of Reproductive Medicine, The First Affiliated Hospital of Xi’an Jiaotong University, 76 West Yanta Road, 710061 Xi’an, Shaanxi People’s Republic of China; 2 Department of Social Medicine and Health Care Management, School of Public Health, Hebei Medical University, 050017 Shijiazhuang, Hebei People’s Republic of China; 3 Hebei Key Laboratory of Environment and Human Health, Hebei Medical University, 050017 Shijiazhuang, Hebei People’s Republic of China; 4 Section of Cancer Stem Cell Research, Key Laboratory of Environment and Genes Related to Diseases, Ministry of Education of the People’s Republic of China, 710061 Xi’an, Shaanxi, People’s Republic of China

**Keywords:** HK2, Fibronectin (FN1), Akt1, Metastasis, Cervical cancer

## Abstract

**Background:**

Hexokinases 2 (HK2) is a member of the hexokinases, linking with malignant tumor growth and distant metastasis. However, evidence regarding the potential role of HK2 in regulating cell motility and tumor metastasis during the cervical cancer malignant progression remains limited.

**Methods:**

In vitro migration and invasion assay, in vivo metastasis experiments were performed to detect the effective of HK2 on regulating cell motility and tumor metastasis in cervical cancer cells. RNA-Seq was performed to explore the potential molecules that participate in HK2-mediated cell motility and tumor metastasis in cervical cancer cells. The correlation between HK2 and Akt1, p-Akt1, FN1 expression in cervical cancer cells and human squamous cervical carcinoma (SCC) samples was verified in this study.

**Results:**

In this study, cervical cancer cells with exogenous HK2 expression exhibited enhanced cell motility and distant metastasis. Transcriptome sequencing analysis revealed that fibronectin (FN1) was significantly increased in HK2-overexpressing HeLa cells, and the PI3K/Akt signaling pathway was identified by KEGG pathway enrichment analysis. Further studies demonstrated that this promotion of cell motility by HK2 was probably a result of it inducing FN1, MMP2 and MMP9 expression by activating Akt1 in cervical cancer cells. Additionally, HK2 expression was altered with the changing of Akt1/p-Akt1 expression, implying that HK2 expression is also modulated by Akt1/p-Akt1. Moreover, the positive correlation between HK2 and Akt1, p-Akt1, FN1 expression in human squamous cervical carcinoma (SCC) samples was verified by using Pearson correlation analysis.

**Conclusions:**

This study demonstrated that HK2 could activate Akt1 in cervical cancer cells, subsequently enhancing cell motility and tumor metastasis by inducing FN1, MMP2 and MMP9 expression. There likely exists an interactive regulatory mechanism between HK2 and Akt1 during the malignant process of cervical cancer.

**Supplementary Information:**

The online version contains supplementary material available at 10.1186/s12935-021-02312-0.

## Background

Cervical cancer is the fourth most common malignant tumor in the world. It is the most common female reproductive system tumor. More than 80% of cases occur in developing countries, and cervical cancer is the second leading cause of cancer-related death among women in developing countries; metastasis of cervical cancer is still the main cause of cancer-related death [1].

Fibronectin (FN1) is one of the most abundant extracellular matrix (ECM) proteins, playing an important role during cell adhesion and migration processes, including during embryogenesis, wound healing, blood coagulation, host defense, and metastasis [2, 3]. FN1 is an established marker for epithelial-mesenchymal transition (EMT), and stimulated FN1 expression has been observed in various types of cancer and is associated with enhanced cell migration, invasion and tumor metastasis [4, 5]. FN1 expression is regulated by a variety of factors (e.g., TGF-β, EGF) and cellular signaling pathways [6, 7]. For instance, the prosurvival effect that FN1 has on cancer cells is primarily mediated by FAK-dependent activation of the PI3K/AKT/mTOR pathway. In cervical cancer, aberrant FN1 expression was confirmed to be involved in cell viability, apoptosis, migration and invasion [8].

HK2 is a member of the hexokinases; it is a pivotal player in the ‘Warburg effect’ and was reported as a key rate-limiting regulator during glucose metabolism [9]. In recent years, HK2 expression has been reported to be elevated in various cancers, facilitating tumor initiation and maintenance, and has been linked to tumor malignant growth [10]. Additionally, the correlation between aberrant HK2 expression and altered cell motility has been confirmed in many types of cancers, including pancreatic cancer [9], neuroblastoma [11], tongue squamous cell carcinoma [10], colon cancer [12], and breast cancer [13]. In cervical cancer, increased HK2 expression in patient samples was reported as early as the 1970s and also confirmed in our previous study, and aberrant expression of HK2 was linked with radiation resistance, malignant cell growth and poor prognosis in cervical cancer [14, 15]. However, evidence about the role of HK2 in regulating cell motility and tumor metastasis during the cervical cancer malignant progression remains limited. In addition, a potential role of HK2 in altering FN1 expression in cancer cells has not been reported.

Therefore, to address these issues, exogenous HK2 was stably overexpressed in cervical cancer cells, and HK2-overexpressing cells exhibited increased cell motility and distant metastasis in cervical cancer cells. Furthermore, a transcriptome sequencing analysis was performed in HK2-overexpressing monoclonal cell lines to screen for potential target genes and signal transduction pathways that are likely involved in HK2-mediated cell migration and tumor metastasis in cervical cancer. As shown in this study, exogenous expression of HK2 activated Akt1 (p-Akt1) in cervical cancer cells, subsequently enhancing cell motility and tumor metastasis by inducing FN1, MMP2 and MMP9 expression.

## Methods

### Cell culture and treatment

The human cervical cancer cell lines (HeLa and SiHa) were purchased from American Type Culture Collection (ATCC; Manassas, VA) and were tested using RT-PCR for mycoplasma contamination every 3 months. The human immortalized cervix squamous cell line (Ect1/E6E7) was purchased from Shanghai GuanDao Biological Engineering Co., Ltd (China). The cervical cancer cell lines HeLa and SiHa were cultured in high-glucose Dulbecco’s modified Eagle’s medium (DMEM, Sigma-Aldrich, St Louis, MO, USA). Ect1/E6E7 cells were cultured in modified Eagle’s medium (MEM, Sigma-Aldrich, St Louis, MO, USA). All of the culture media described above were mixed with 10% FBS (fetal bovine serum, HyClone, Thermo Scientific, Waltham, MA, USA). HK2-overexpressing cells were treated with 5 µM MK2206 (an inhibitor of Akt1, Selleck, S1078, USA), as previously described [16]. Then, the cells were subjected to western blot, migration and invasion assays.

### Plasmid construction and transfection

The pIRES2-AcGFP-HK2 plasmid was constructed using the following primers: forward, 5′-CCGGAATTCGCCACCATGATTGCCTCGCATCTGCTTGCCTACT-3′, and reverse, 5′-CGCGGATCCCTATCGCTGTCCAGCCTCACGGATGC-3′. The primers were used to amplify the full-length human HK2 coding sequence and cloned into the pIRES2-AcGFP vector (Clontech, Mountain View, CA) via the EcoRI and BamHI sites. The shRNA for HK2 was purchased from Gene Pharma (Shanghai, China). The pIRES2-AcGFP-HK2 and shRNA vectors were transfected into SiHa and HeLa cells with Lipofectamine 2000 reagent (Invitrogen, Carlsbad, CA, USA), and then the cells were treated with G418 (Calbiochem, La Jolla, CA, USA) for three-four weeks to generate stably overexpression and knockdown cell lines. pIRES2-AcGFP-Akt1 was a gift of Dr. Na Cai from our laboratory [17] and was transiently transfected into cells by using Lipofectamine 2000 reagent.

### Western blotting

The western blotting analysis used in this study was performed as previously described [16]. Horseradish peroxidase-conjugated anti-rabbit or anti-mouse IgG was purchased from Thermo Fisher Scientific (New York, NY, USA). The antibodies used were as follows: anti-HK2 (1:500 dilution, sc-374091, Santa Cruz, USA), anti-Akt1 (1:500 dilution, sc-5298, Santa Cruz, USA), anti-p-Akt1 (1:500 dilution, sc-293125, Santa Cruz, USA), anti-GAPDH (1:500 dilution, sc-47724, Santa Cruz, USA), anti-MMP2 (1:1,000 dilution, 10373-2-AP, Wuhan, China), anti-MMP9 (1:1,000 dilution, 10375-2-AP, Wuhan, China), and anti-FN1 (1:1,000 dilution, 15613-1-AP, Wuhan, China). GAPDH was used as the control and for quantification.

### In vitro migration and invasion assays

For the wound-healing assay in vitro, cervical cancer cells stably transduced with HK2 were plated in 6-well plates and cultured in DMEM containing 10% FBS. When the cells grew to nearly 100% confluence, the cell monolayers were scratched by using a pipette tip and washed with PBS and then cultured in DMEM without FBS at 37 °C. Then, cell monolayers were photographed by using a digital camera mounted on an inverted microscope at 0, 24 h and 48 h. The wound area was measured using ImageJ software, and the migration potential was calculated according to the equation: wound scratch area = (wound scratch area at 0 h)—(wound scratch area at 48 h or 72 h). Three independent experiments were performed for wound-healing assay.

For the migration and invasion assays in vitro, cells (4 × 10^4^ cells) stably transduced with HK2 recombinant plasmid, transiently transfected with Akt1 recombinant plasmid or treated with MK2206 were added to upper transwell chambers with or without Matrigel (BD Biosciences, San Jose, CA) and incubated for 48 h. The medium in the upper chambers contained 1% FBS, and the medium in the lower chambers contained 10% FBS. The cells that migrated and invaded through the Matrigel-uncoated or -coated membranes were permeabilized with 70% methanol and stained with 0.1% crystal violet. Five fields for every chamber were photographed and counted by a scientist blinded to the experimental conditions. Three independent experiments were performed for the migration or invasion assays.

### In vivo metastasis experiments in BALB/c nude mice

The experimental protocols were evaluated and approved by the Animal Care and Use Committee of the Medical School of Xi’an Jiaotong University, and all of the animals were raised in a specific pathogen-free (SFP) room, with constant temperature (22–25 °C) and humidity (40–50%). For the in vivo metastasis experiments, twenty-four 6- to 7-week-old female BALB/c-nude mice (purchased from SLAC Laboratory Animal Co., Ltd., Shanghai, China) were randomly divided into four groups, 5 × 10^5^ HeLa-HK2 or SiHa-HK2 cells and control cells were injected via the tail vein of each mouse, 10 × 10^5^ HeLa-shHK2 and HeLa-shCtr cells were injected via the tail vein of each mouse. The mice were observed every three days after injection, no death was observed in the two groups. After two to three months, the mice were euthanised with carbon dioxide, and the lungs and livers were removed and subjected to histologic examination.

### ELISA analysis

The cell culture media was collected, the expression of MMP2 and MMP9 in cell medium supernatant was detected by the enzyme-linked immunosorbent assay (ELISA).

The detailed procedures were conducted strictly with the protocol in the Quantikine® ELISA human MMP2 (MMP200) and MMP9 (DMP900) immunoassay kit (R&D system, Minneapolis, MN, USA).

### Real time PCR analysis

Total RNA extraction and the protocol for real-time PCR were performed as previously described [16]. GAPDH was used as the housekeeping gene in this study, and all of the results were analyzed via the ∆∆Ct method. The primer sequences that used in this study for real time PCR were as follows: FN1 (F: 5-′CGGTGGCTGTCAGTCAAAG-3′R: 5-′AAACCTCGGCTTCCTCCATAA-3′), GAPDH (F: 5-′CACCGTCAAGGCTGAGAAC-3′ and 5-′TGGTGAAGACGCCAGTGGA-3′).

### RNA preparation and transcriptome resequencing

Total RNA of HeLa-Vec and HeLa-HK2 monoclonal cells was extracted by using TRIzol reagent (Invitrogen, Carlsbad, CA, USA) for transcriptome resequencing. Samples were analyzed using the BGISEQ-500 platform (The Beijing Genomics Institute, BGI), and the average output of each sample was 1.15 Gb. The average ratio of sample to genome was 94.94%, and the ratio of comparison to each gene set was 79.16%. The experimental analysis used the NOISeq method, which is a novel nonparametric approach for the identification of differentially expressed genes (DEGs) based on thresholds of log2 fold change > 1 and a probability ≥ 0.80. Subsequent data analysis was performed online by Dr. Tom from the Beijing Genomics Institute. The transcriptome data were uploaded on https://www.ncbi.nlm.nih.gov/bioproject/PRJNA670405.

### Immunohistochemistry assay

The human squamous cervical carcinoma (SCC) samples used in this study were collected at the First Affiliated Hospital of Xi’an Jiaotong University from 2008 to 2016 as described in our previous study. The immunohistochemical staining procedure and the evaluation standard of immunohistochemistry (IHC) score were performed as previously described [18]. To validate the correlation between HK2 and Akt1, p-Akt1, FN1 expression in vivo, serial sections of human squamous cervical carcinoma samples (n = 15) were immunostained with an anti-HK2 (1:100 dilution, sc-374091, Santa Cruz, USA), anti-Akt1 (1:100 dilution, sc-5298, Santa Cruz, USA), anti-p-Akt1 (1:100 dilution, sc-293125, Santa Cruz, USA), anti-FN1 (1:300 dilution, 15613-1-AP, Wuhan, China). The immunohistochemistry (IHC) score of HK2, Akt1, p-Akt1 and FN1 in these SCC samples was confirmed by using Pearson correlation analysis.

### Statistical analysis

All of statistical analysis in this study was performed with Graphpad Prism 8.0 software and SPSS software version 19.0. For comparison among groups, the χ^2^ test or one-way ANOVA was performed. Two-tailed unpaired Student’s t-test was used to determine the statistical significance for 2-group analyses, and presented as mean ± SD. Post hoc test was performed for comparison among groups. In all of the tests, statistical significance was defined as *p* < 0.05.

## Results

### HK2 promotes cell migration and invasion of cervical cancer cells in vitro

**Fig. 1:**
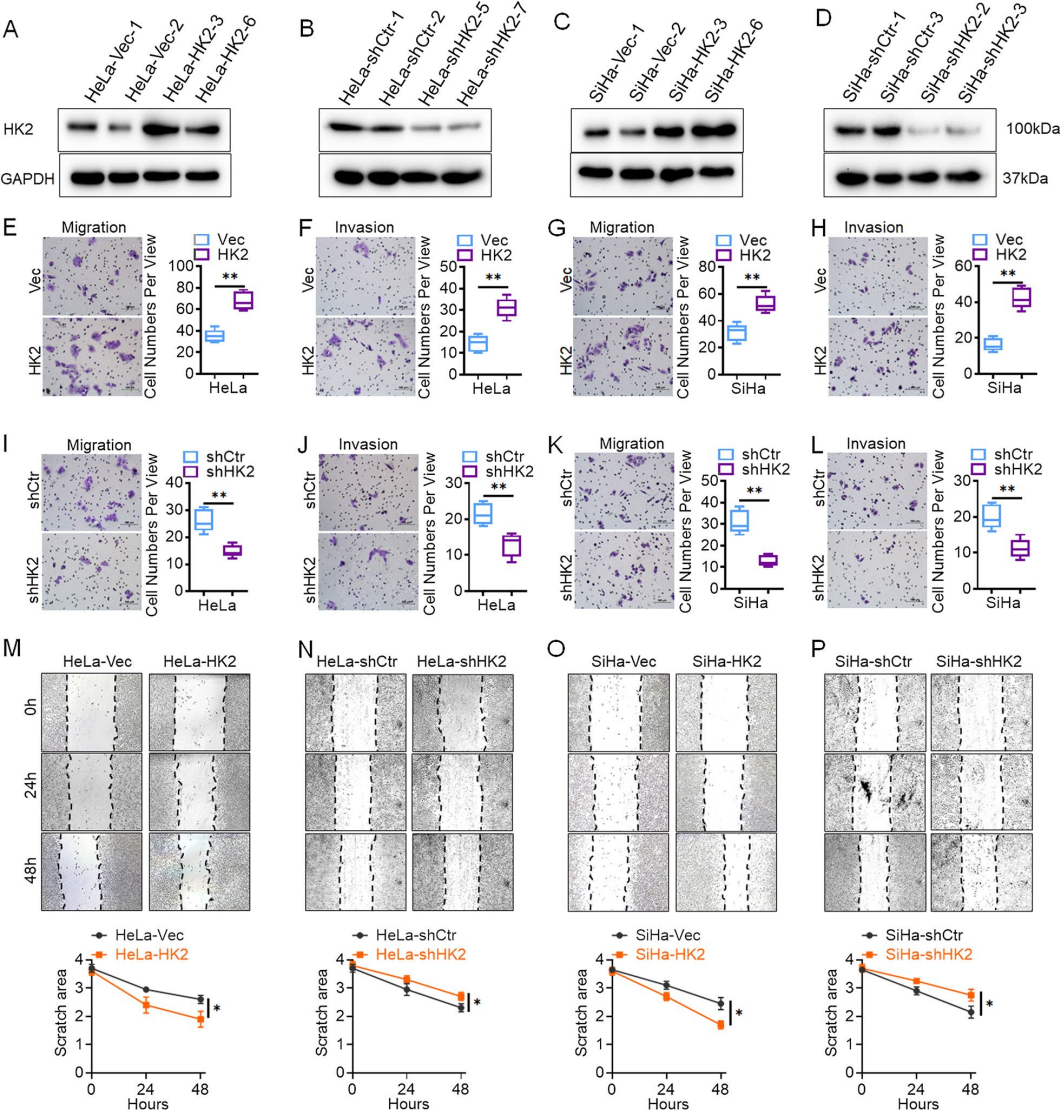
HK2 enhances the migration and invasion ability of SiHa and HeLa cells in vitro. Stably transfected cell lines were identified by western blotting: **A** HeLa-Vec and HeLa-HK2 cells; **B** HeLa-shControl and HeLa-shHK2 cells. **C** SiHa-Vec and SiHa-HK2 cells; **D** SiHa-shControl and SiHa-shHK2 cells. The migratory capacities were analyzed by the transwell assay, and the number of migratory cells is shown (scale bar, 100 μm). **E** HeLa-Vec and HeLa-HK2 cells **G** SiHa-Vec and SiHa-HK2 cells. **I** HeLa-shCtr and HeLa-shHK2 cells **K** SiHa-shCtr and SiHa-shHK2 cells. The invasive capacities were analyzed by the transwell assay, and the number of migratory cells is shown (scale bar, 100 μm). **F** HeLa-Vec and HeLa-HK2 cells **H** SiHa-Vec and SiHa-HK2 cells. **J** HeLa-shCtr and HeLa-shHK2 cells **L** SiHa-shCtr and SiHa-shHK2 cells. The migratory potential was analyzed by wound-healing assays performed for 0, 24, and 48 h. **M** HeLa-Vec and HeLa-HK2 cells **N** HeLa-shCtr and HeLa-shHK2 cells **O** SiHa-Vec and SiHa-HK2 cells. **P** SiHa-shCtr and SiHa-shHK2 cells (scale bar, 200 μm). The data are shown as the mean ± SD of three independent experiments. *p < 0.05, **p < 0.01 vs. control using one-way ANOVA

Data from the GEPIA online database (http://gepia.cancer-pku.cn/) revealed that HK2 expression was much higher in cervical cancer tissues than in normal tissues (Additional file 1: Fig. S1A), and such increased HK2 was associated with poor prognosis in cervical cancer patients (Additional file 1: Fig. S1B). In our previous study, a relatively low expression of HK2 was observed in HeLa and SiHa cells among five cervical cancer lines (HeLa, SiHa, C-33 A, CaSki and HT-3) [15]. Subsequently, to further investigate the function of HK2 on regulating cell motility and tumor metastasis in human cervical cancer cells, exogenous HK2 was stably overexpressed by transfection of an HK2 recombinant plasmid in HeLa (HeLa-Vec and HeLa-HK2, Fig. 1A) and SiHa (SiHa-Vec and SiHa-HK2, Fig. 1C) cells. Endogenous HK2 was knocked down by using two of efficiently HK2 shRNA vectors (shHK2-1962 and shHK-2207) in HeLa (HeLa-shControl and HeLa-shHK2, Fig. 1B) and SiHa (SiHa-shControl and SiHa-shHK2, Fig. 1D) cells, respectively.

Wound-healing assays and transwell assays were employed to evaluate the capacity for cell motility in HK2-modified cervical cancer cells and control cells. After incubation for 48 h, transwell migration analysis revealed that the numbers of HeLa-HK2 (67.80 ± 8.41, *p* < 0.05, Fig. 1E) and SiHa-HK2 (52.60 ± 6.19, *p* < 0.05, Fig. 1G) cells that migrated across the uncoated membrane were much higher than those of HeLa-Vec (35.2 ± 5.85) and SiHa-Vec (31.20 ± 6.27) cells. Conversely, the numbers of cells that migrated across the uncoated membrane were much lower in HK2-knockdown HeLa (14.80 ± 2.28, *p* < 0.05, Fig. 1I) and SiHa (12.80 ± 2.59, *p* < 0.05, Fig. 1K) cells than in HeLa-shControl (26.20 ± 4.21) and SiHa-shControl (31.00 ± 5.34) cells. Similarly, after incubation for 48 h, a significant increase in wound closure was found in HeLa-HK2 (*p* < 0.05, Fig. 1M) and SiHa-HK2 (*p* < 0.05, Fig. 1O) cells compared with that observed in the respective control cells (HeLa-Vec and SiHa-Vec cells, respectively). Conversely, a significant decrease in wound closure was found in HeLa-shHK2 (*p* < 0.05, Fig. 1N) and SiHa-shHK2 (*p* < 0.05, Fig. 1P) cells compared with that observed in the respective control cells (HeLa-shControl and SiHa-shControl cells, respectively). These results demonstrate that exogenously expressed HK2 in HeLa and SiHa cells significantly enhances cell migratory capacity in vitro.

Furthermore, the transwell membrane was coated with Matrigel, and of the changes in invasive capacity of HK2-modified HeLa and SiHa cells were detected. The numbers of HeLa-HK2 (31.00 ± 4.48, *p* < 0.05, Fig. 1F) and SiHa-HK2 (42.20 ± 5.76, *p* < 0.05, Fig. 1H) cells that migrated across the coated membrane were much greater than those of HeLa-Vec (14.40 ± 3.85) and SiHa-Vec (16.20 ± 3.71) cells. Conversely, the numbers of cells that migrated across the Matrigel-coated membrane were much lower in HK2 knockdown HeLa (12.80 ± 3.27, *p* < 0.05, Fig. 1J) and SiHa (11.20 ± 2.59, *p* < 0.05, Fig. 1L) cells than in HeLa-shControl (21.00 ± 3.05) and SiHa-shControl (20.00 ± 3.39) cells. These results demonstrate that exogenously expressed HK2 in HeLa and SiHa cells enhances the invasive capacity of HeLa and SiHa cells in vitro.

### HK2 promotes distant metastasis in cervical cancer in vivo

**Fig. 2:**
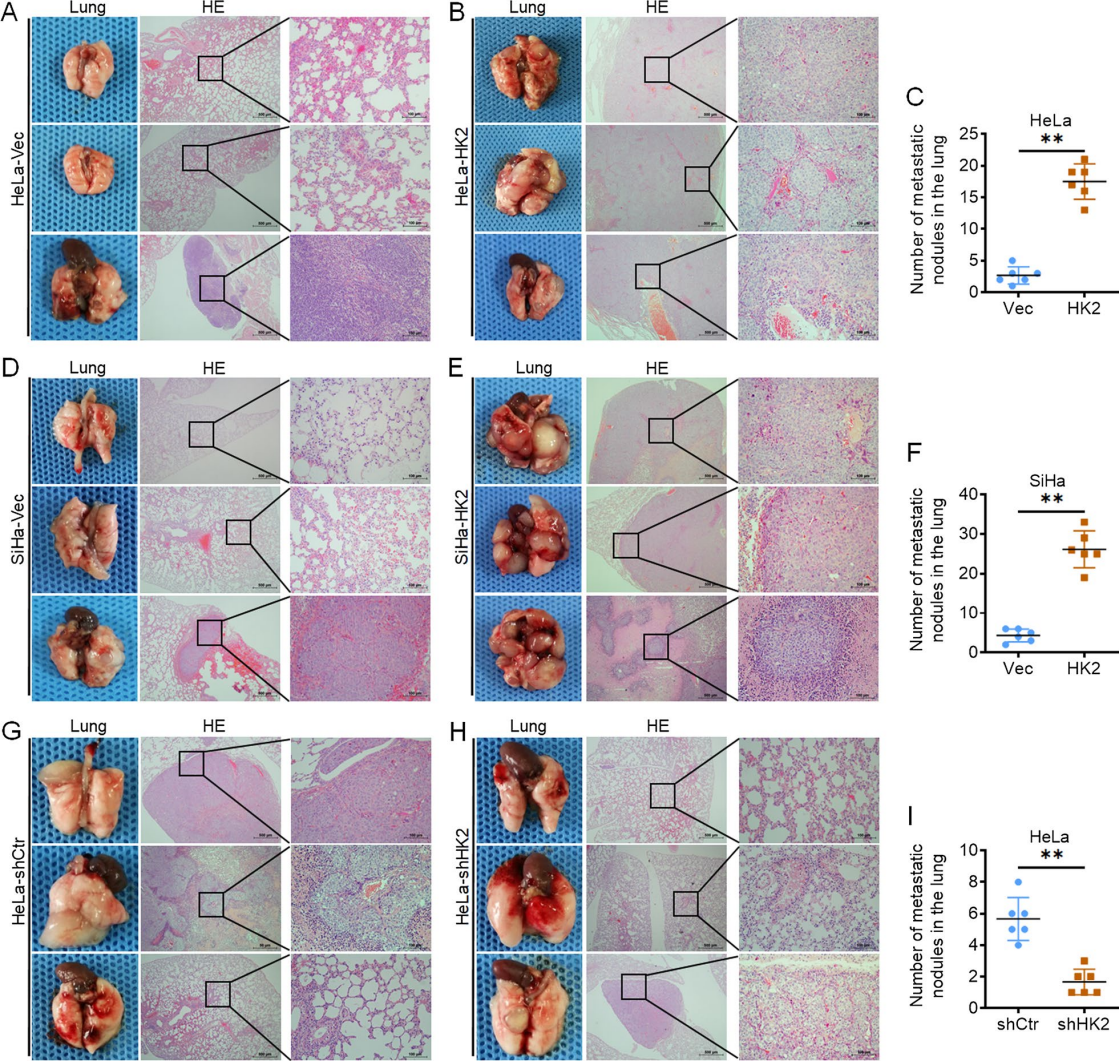
HK2 overexpression promotes distant metastasis of SiHa and HeLa cells in vivo. Female BALB/c nude mice were injected via the tail vein with HeLa-Vec and HeLa-HK2 cells. Representative lung and hematoxylin and eosin-stained images are presented to show tumor lesions in the HeLa-Vec (**A**) and HeLa-HK2 (**B**) groups. **C** The scatter plots show the number of lesions in the lungs as the mean ± SD (n = 6). Female BALB/c nude mice were injected via the tail vein with SiHa-Vec and SiHa-HK2 cells. Representative lung and hematoxylin and eosin-stained images are presented to show tumor lesions in the SiHa-Vec (**D**) and SiHa-HK2 (**E**) groups. **F** The scatter plots show the number of lesions in the lungs as the mean ± SD (n = 6). Female BALB/c nude mice were injected via the tail vein with HeLa-shCtr and HeLa-shHK2 cells. Representative lung and hematoxylin and eosin-stained images are presented to show tumor lesions in the HeLa-shCtr (**G**) and HeLa-shHK2 (**H**) groups. **I** The scatter plots show the number of lesions in the lungs as the mean ± SD (n = 6). Scale bars, 500 μm and 100 μm. Data were statistically analyzed with Student’s t-test, and values are shown as the mean ± SD. **p < 0.01

To further identify the effect of HK2 on distant metastasis in vivo, 5 × 10^5^ HeLa-HK2 or SiHa-HK2 cells and control cells were injected into female nude mice via the tail vein. Organ metastases in nude mice were observed after two to three months. The metastatic tumor lesions in the HeLa-HK2 or SiHa-HK2 group and control groups were counted under microscopy by H&E staining. As shown in Fig. 2A and B, the metastatic tumors in lung tissues derived from HeLa-HK2 cells were much more numerous and larger than those derived from HeLa-Vec cells, and the average number of metastatic lesions counted under microscopy was much greater in the HeLa-HK2 group (17.50 ± 2.81, *p* < 0.01, Fig. 2C) than in the HeLa-Vec group (2.67 ± 1.36). Similarly, the metastatic tumors in lung tissues derived from SiHa-HK2 cells were much more numerous and larger than those derived from the SiHa-Vec cells (Fig. 2D, E), and the average number of metastatic lesions counted under microscopy was much greater in the SiHa-HK2 group (26.17 ± 4.67, *p* < 0.01, Fig. 2F) than in the SiHa-Vec group (4.33 ± 1.64). Conversely, the metastatic tumors in lung tissues derived from HeLa-shHK2 cells were much less numerous and smaller than those derived from HeLa-shCtr cells (Fig. 2G, H), and the average number of metastatic lesions counted under microscopy was much less in the HeLa-shHK2 group (1.65 ± 0.82, *p* < 0.01, Fig. 2I) than in the HeLa-shCtr group (5.67 ± 1.36). Regrettably, no metastatic nodules were observed in the liver in mice injected with HeLa-HK2 or SiHa-HK2 cells and control cells. These results demonstrated that HK2 protein facilitates the lung metastasis of HeLa and SiHa cells in vivo.

### HK2 activates FN1 and Akt1/p-Akt1 expression in cervical cancer cells

**Fig. 3:**
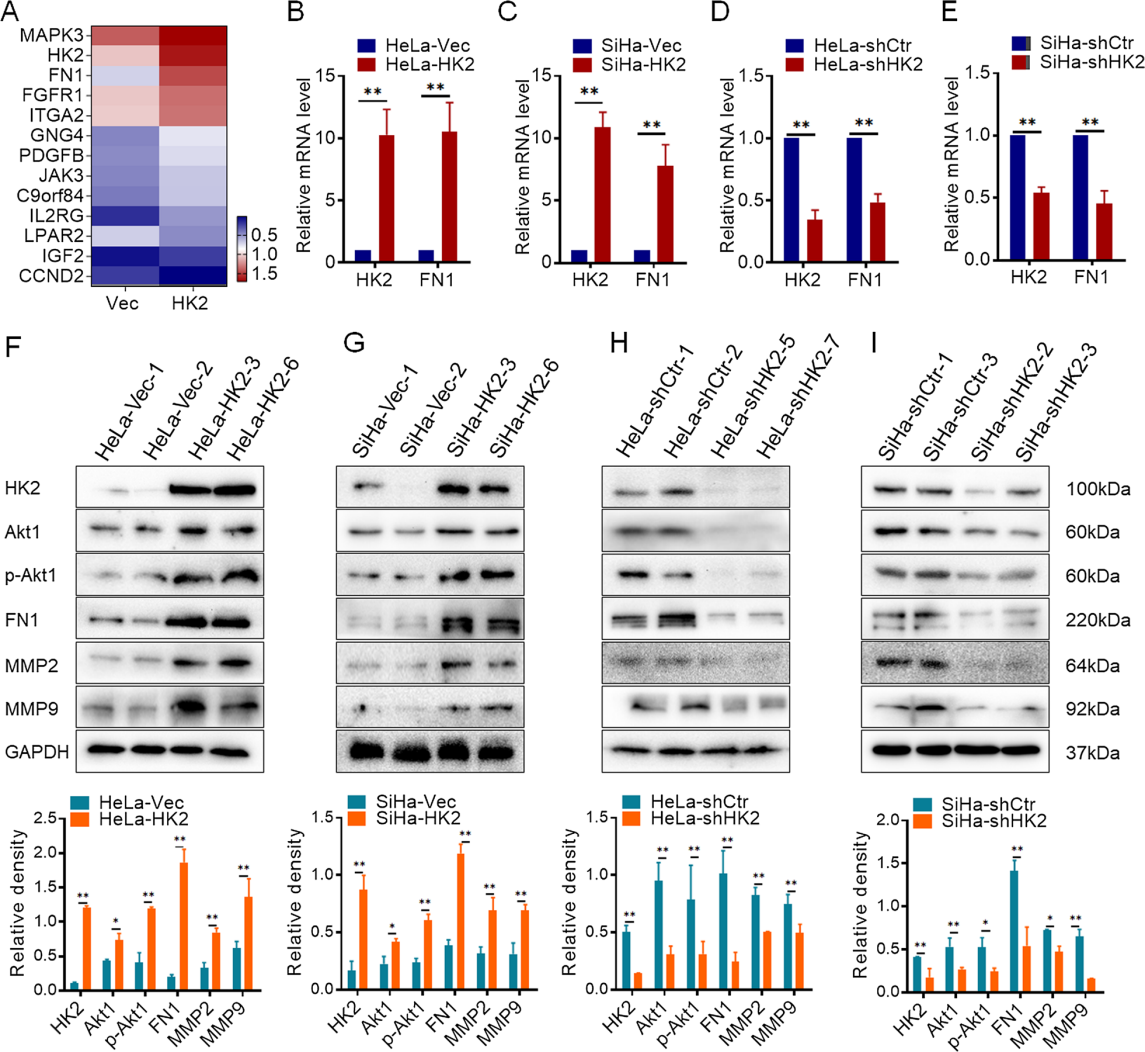
HK2 overexpression induces FN1 expression and activates Akt1/p-Akt1. **A** Heatmap of the enriched differentially expressed genes in the PI3K/Akt signaling pathway identified by GO enrichment analysis of RNA transcriptome sequencing data from HeLa-HK2 and HeLa-Vec cells; data were log^10^ normalized. **B** The mRNA expression of FN1 was detected by real-time quantitative PCR, and the quantitative analysis is shown: **B** HeLa-Vec and HeLa-HK2 cells; **C** SiHa-Vec and SiHa-HK2 cells; **D** HeLa-shControl and HeLa-shHK2 cells. **E** SiHa-shControl and SiHa-shHK2 cells. The protein levels of HK2, Akt1, p-Akt1, FN1, MMP2 and MMP9 were detected by western blotting, and the quantitative analysis is shown: **F** HeLa-Vec and HeLa-HK2 cells; **G** SiHa-Vec and SiHa-HK2 cells; **H** HeLa-shControl and HeLa-shHK2 cells; **I** SiHa-shControl and SiHa-shHK2 cells. The data are shown as the mean ± SD of three independent experiments. *p < 0.05, **p < 0.01 vs*.* control using one-way ANOVA

To further explore the potential molecular mechanism by which HK2 promotes the migratory and invasive abilities of cervical cancer cells, transcriptome sequencing analysis was performed in HeLa-HK2 and HeLa-Vec monoclonal cell lines. A total of 210,503 transcripts were detected, and 258 upregulated and 152 downregulated genes were identified between the HeLa-HK2 and HeLa-Vec groups. The PI3K/Akt signaling pathway was identified by KEGG pathway enrichment analysis and included 12 identified genes (Fig. 3A). Unexpectedly, *fibronectin* (*FN1*), a key factor associated with cancer cell differentiation, growth, adhesion, migration, and invasion [19–21], was one of the 12 identified genes and was significantly increased in the HeLa-HK2 group. Therefore, the mRNA level of *fibronectin* (*FN1*) in HK2-modified cells was confirmed by real-time PCR. As shown in Fig. 3B and C, the mRNA level of FN1 was increased in both HK2-overexpressing cells (HeLa-HK2 and SiHa-HK2) and decreased in HK2-knockdown cells (SiHa-shHK2 and HeLa-shHK2, Fig. 3D and E, p < 0.05). Consistent with the mRNA results, the protein level of FN1 was also increased in both HK2-overexpressing cells (SiHa-HK2 and HeLa-HK2, Fig. 3F and G, p < 0.05) and decreased in HK2-knockdown cells (SiHa-shHK2 and HeLa-shHK2, Fig. 3H and I, p < 0.05).

Although the pivotal role of FN1 in regulating cell motility and tumor metastasis has been reported in numerous studies, there are still few reports about the relationship between HK2 and FN1 in cancer cells. According to current research, both HK2 and FN1 are linked with the PI3K/Akt signaling pathway [22, 23], and the KEGG pathway enrichment analysis in this study also indicated that the PI3K/Akt signaling pathway was altered in HK2-overexpressing HeLa cells. Therefore, Akt1 and p-Akt1 [24], the key factors in the PI3K/Akt signaling pathway regulating cellular processes (e.g., cell proliferation, differentiation, migration and survival), were detected by western blot in HK2-modified cells. As shown in Fig. 3, the protein levels of both Akt1 and p-Akt1 were upregulated in HeLa-HK2 cells (Fig. 3F, p < 0.01) and SiHa-HK2 cells (Fig. 3G, p < 0.01) and downregulated in HK2-knockdown cells (HeLa-shHK2, Fig. 3H, p < 0.01 and SiHa-shHK2, Fig. 3I, p < 0.01). Additionally, the mRNA level of Akt1 was increased in both HK2-overexpressing cells (HeLa-HK2 and SiHa-HK2, Additional file 1: Fig. S1C, *p* < 0.05). Moreover, the p-Akt1/Akt1 ratio was increased in HK2-overexpressing cells (HeLa-HK2 and SiHa-HK2, *p* < 0.05, Additional file 1: Table S1) and decreased in HK2 knocked down cells (HeLa-shHK2 and SiHa-shHK2, *p* < 0.05, Additional file 1: Table S1), comparing with their control cells. These results suggest that HK2 induces FN1 expression and activates Akt1 (p-Akt1) in cervical cancer cells.

Additionally, FN1 was shown to induce MMP2 [25, 26] and MMP9 [27] expression during the malignant progression of human cancers in previous studies, which was conducive to tumor migration, invasion, angiogenesis, and intravasation [28]. As shown in Fig. 3, the protein levels of both MMP2 and MMP9 were upregulated in HK2-overexpressing cells (HeLa-HK2, Fig. 3F, p < 0.01; and SiHa-HK2, Fig. 3G, p < 0.01) and downregulated in HK2-knockdown cells (HeLa-shHK2, Fig. 3H, p < 0.01; and SiHa-shHK2, Fig. 3I, p < 0.01). Moreover. the expression of MMP2 and MMP9 in cell medium supernatant was detected by the enzyme-linked immunosorbent assay (ELISA). As shown in Additional file 1: Fig. S1G, the protein levels of MMP2 and MMP9 in cell medium supernatant were increased in both HeLa-HK2 and SiHa-HK2 cells, comparing with their control cells (Additional file 1: Fig. S1G, *p* < 0.05). Conversely, the protein levels of MMP2 and MMP9 in cell medium supernatant were decreased in HK2-knockdown cells (HeLa-shHK2 and SiHa-shHK2, Additional file 1: Fig. S1G, *p* < 0.05).

Additionally, an HK2 recombinant plasmid was transient transfected in Etc1/E6E7 cells (an immortalized human cervix squamous cell line). As shown in Additional file 1: Fig. S1H, increased HK2, Akt1, p-Akt1, FN1, MMP2 and MMP9 expression was observed in Etc1/E6E7-HK2 cells (*p* < 0.05), and these was accompanied with enhanced cell migratory and invasive capacities in Etc1/E6E7-HK2 cells (Additional file 1: Fig. S1I, *p* < 0.05). All of these results demonstrate that stimulated HK2 expression induces Akt1 (p-Akt1), FN1, MMP2 and MMP9 expression in cervical cancer cells.

### Blocking Akt1/p-Akt1 in HK2-overexpressing cells inhibits the stimulation of cell motility

**Fig. 4:**
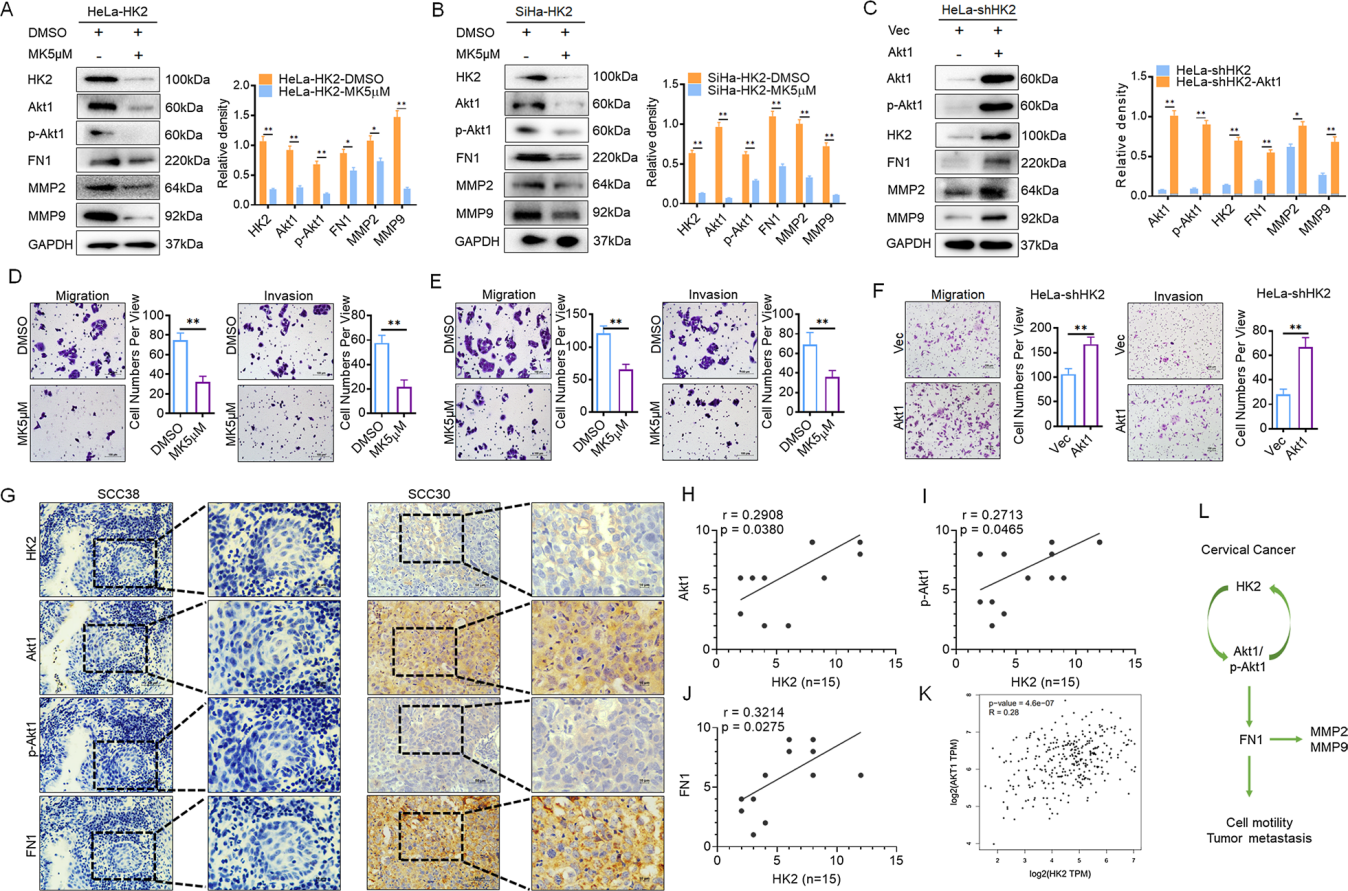
Alteration of Akt1/p-Akt1 expression in HK2-modified cells. The protein levels of Akt1 and p-Akt1 in HeLa-HK2 and SiHa-HK2 cells were inhibited by the Akt1 inhibitor MK2206. The protein levels of Akt1, p-Akt1, HK2, FN1, MMP2 and MMP9 were detected by western blotting in HeLa-HK2 (**A**) and SiHa-HK2 (**B**) cells, and quantitative analysis is shown. **C** The protein levels of Akt1, p-Akt1, HK2, FN1, MMP2 and MMP9 in HeLa-shHK2 cells transiently transfected with an Akt1 recombinant plasmid were detected by western blotting, and quantitative analysis is shown. The migratory and invasive capacities of MK2206-treated HeLa-HK2 (**D**) and SiHa-HK2 (**E**) cells were analyzed by the transwell assay, and the number of cells is shown (scale bar, 100 μm). **F** An Akt1 recombinant plasmid was transiently transfected into HeLa-shHK2 cells, the migratory and invasive capacities were analyzed by the transwell assay. **G** The expression of HK2, Akt1, p-Akt1 and FN1 was detected in serial sections of SCC samples by using immunocytochemistry analysis (scale bar, 50 and 10 μm). The correlation between Slug and Akt1(**H**), p-Akt1 (**I**), FN1 (**J**), in SCC samples was confirmed by using Pearson correlation analysis, n = 15. **K** The positive correlation between HK2 and Akt1 expression in cervical squamous cell carcinoma and endocervical adenocarcinoma (CESC) was confirmed from the GEPIA online database. **L** Proposed model of the mechanisms by which HK2 promotes motility and distant metastasis in cervical cancer. The data are shown as the mean ± SD of three independent experiments. *p < 0.05, **p < 0.01 vs. control using one-way ANOVA

To further confirm that the stimulation of Akt1 (p-Akt1) in HK2-overexpressing cells was responsible for the induced FN1 expression in this study, a pan Akt kinase inhibitor (MK2206) was used to block induced Akt1/p-Akt1 expression in HeLa-Vec, HeLa-HK2, SiHa-Vec and SiHa-HK2 cells. As shown in Fig. 4 and Additional file 1: Fig. S1, reduced Akt1, p-Akt1, FN1, MMP2 and MMP9 expression was observed in MK2206-treated HeLa-HK2 (Fig. 4A, p < 0.05), HeLa-Vec (Additional file 1: Fig. S1D, *p* < 0.05), SiHa-HK2 (Fig. 4B, p < 0.05) and SiHa-Vec cells (Additional file 1: Fig. S1E, *p* < 0.05) and this was accompanied by inhibited cell migratory and invasive capacities in HeLa-HK2 (Fig. 4D, p < 0.05) and SiHa-HK2 cells (Fig. 4E, p < 0.05). Additionally, Akt1/p-Akt1 expression was rescued in HK2-knockdown HeLa-shHK2 cells (Fig. 4C, p < 0.05) and control cells (Additional file 1: Fig. S1F) via transient transfection of an Akt1 recombinant plasmid (pIRES2-AcGFP-Akt1). As shown in Fig. 4C, Akt1/p-Akt1 expression was recovered, FN1, MMP2, and MMP9 expression was increased (*p* < 0.05); cells exhibited enhanced migration and invasion (Fig. 4F, p < 0.05). All of these results further confirm that HK2 induces FN1, MMP2, and MMP9 expression by stimulating Akt1 (p-Akt1) in cervical cancer cells, subsequently enhancing cell motility.

To validate positive correlation between the expression of HK2 and Akt1, p-Akt1, FN1 in cervical cancer in vivo, serial sections of human squamous cervical carcinoma (SCC) samples (n = 15) were immunostained with antibodies specific for HK2, Akt1, p-Akt1, and FN1 (Fig. 4G). Furthermore, the positive correlation between HK2 and Akt1 (Fig. 4H, r = 0.2908, *p* = 0.0380), p-Akt1 (Fig. 4I, r = 0.2713, *p* = 0.0465), FN1 (Fig. 4J, r = 0.3214, *p* = 0.0275) expression in these SCC samples was confirmed by using Pearson correlation analysis. Additionally, the positive correlation between HK2 and Akt1 expression in cervical squamous cell carcinoma and endocervical adenocarcinoma (CESC) was confirmed from the GEPIA online database (Fig. 4K).

Interestingly, when the induction of Akt1 (p-Akt1) was blocked by MK2206 in HK2-overexpressing HeLa-HK2 (Fig. 4A, p < 0.05) and SiHa-HK2 cells (Fig. 4B, p < 0.05)), the protein level of HK2 was also significantly decreased. Conversely, when Akt1 (p-Akt1) expression was rescued via transfection of an Akt1 recombinant plasmid, HK2 expression was recovered and significantly increased in HeLa-shHK2 cells (Fig. 4C, p < 0.05). These results implied that there is likely crosstalk between HK2 and Akt1 (p-Akt1) regulating their expression during malignant progression in cervical cancer (Fig. 4L).

## Discussion

The data from the GEPIA online database (http://gepia.cancer-pku.cn/) revealed that HK2 expression was much higher in cervical cancer tissues than in normal tissues, and high expression of HK2 was associated with poor outcome in cervical cancer patients. Consistent with previous studies that confirmed increased HK2 expression in cervical cancer specimens [29], our previously study demonstrated that the positive rate of HK2 was much higher in cervical cancer samples than normal cervix samples [15]. Although the promoting effect of HK2 on malignant cell growth and tumor metastasis has been reported in many types of cancers [30–32], the potential role of HK2 in regulating cell motility and tumor metastasis in cervical cancer cells remains unclear. In this study, exogenously expressed HK2 in HeLa and SiHa cells significantly enhanced cell motility in vitro and promoted distant metastasis in vivo. When HK2-overexpressing HeLa and SiHa cells were injected into female nude mice via the tail vein, the metastatic tumors in lung tissues were much larger than those in the control group. All of these results demonstrate that exogenously expressed HK2 enhances cell motility in vitro and promotes distant metastasis in *vivo*. This promoting effect of HK2 on cell motility and distant metastasis in cervical cancer cells was consistent with that found in other cancers [9–11].

To explore the potential regulatory mechanism by which HK2 regulates cell motility and invasion in cervical cancer cells, transcriptome sequencing analysis was performed in HeLa cells to screen for potential target genes or signaling pathways mediated by HK2. Accumulated evidence has confirmed a close connection between HK2 and the PI3K/Akt signaling pathway during abnormal glycometabolic processes and tumor malignant development [33, 34]. As expected, the PI3K/Akt signaling pathway was identified by KEGG pathway enrichment analysis in HeLa-HK2 cells, including 12 significantly different genes. Unexpectedly, *fibronectin* (*FN1*), a key factor involved in cell motility and tumor metastasis [35], was one of these 12 identified genes and was significantly increased in HeLa-HK2 cells. Moreover, the increased mRNA and protein levels of fibronectin (FN1) were confirmed in HK2-overexpressing HeLa and SiHa cells. However, the molecular mechanism of interaction between HK2 and FN1 in cervical cancer remains unknown. Therefore, we supposed that such stimulation of FN1 in HK2-overexpressing cells was probably responsible for the promoting effect of HK2 on cell motility and tumor metastasis in cervical cancer.

In recent studies, the activation of the PI3K/Akt signaling pathway has been shown to be instrumental for FN1 transcription and alternative splicing, modulating cell behavior in various cancers [8]. The interactive relationship between the PI3K/Akt signaling pathway and HK2 has also been confirmed in various cancers [36]. Therefore, we hypothesized that the PI3K/Akt signaling pathway served as a mediator between HK2 and FN1 in this study. As previous studies reported, Akt1 is a crucial downstream effector of the PI3K/Akt signaling pathway and is involved in the regulation of cellular activities [8, 37, 38]. In this study, the protein levels of both Akt1 and p-Akt1 were significantly increased in HK2-overexpressing cells but reduced in HK2-knockdown cells, suggesting that the protein level of Akt1 (p-Akt1) was positively correlated with HK2 in cervical cancer cells. When Akt1/p-Akt1 expression was blocked in SiHa-HK2 and HeLa-HK2 cells by using an Akt inhibitor (MK2206), the increased FN1 expression was inhibited, and impaired cell motility resulted. Conversely, FN1 expression was recovered when Akt1/p-Akt1 expression was rescued by transient transfection of an Akt1 recombinant plasmid in HeLa-shHK2 cells, which then exhibited enhanced motility. These results suggest that the induction of FN1 in HK2-overexpressing cervical cancer cells must be a result of the activation of Akt1 (p-Akt1). However, as a pan Akt kinase inhibitor, when the MK2206 used to inhibit the expression of Akt1 (p-Akt1), it could not exclude the potential effect of Akt2 and Akt3 on regulating FN1. The specific isoform of Akt (potential effect of Akt2 or Akt3) that involved in this process still need more experiments to conform in future study.

FN1 was previously shown to be able to activate MMP2 [25, 26] and MMP9 [27] expression during malignant tumor progression, facilitating cell migration, invasion, and distant metastasis [28]. Consistently, the protein levels of MMP2 and MMP9 were increased in HK2-overexpressing cells but reduced in HK2-knockdown cells. When the increased FN1 expression was inhibited by treatment with MK2206 in HK2-overexpressing cells, MMP2 and MMP9 expression was also reduced, and diminished cell motility resulted. Conversely, the protein level of MMP2/MMP9 and cell motility were recovered when FN1 expression was rescued by transient transfection of an Akt1 recombinant plasmid in HK2-knockdown cells, suggesting that there is probably an HK2-Akt1-FN1-MMP2/MMP9 signaling axis promoting cell motility in cervical cancer cells. All of these results demonstrate that HK2 promotes cell motility, suggesting that HK2 probably facilitates tumor malignant growth of metastases.

Interestingly, although a significantly increased protein level of Akt1/p-Akt1 was observed in HK2-overexpressing cervical cancer cells, when such elevated Akt1/p-Akt1 expression was inhibited by using MK2206, the protein level of HK2 was also significantly diminished in HK2-overexpressing cells. The protein level of HK2 was rescued in HK2-knockdown cells when Akt1/p-Akt1 expression was recovered by transient transfection of an Akt1 recombinant plasmid. These results imply that HK2 expression can be mediated through the activation of Akt1 (p-Akt1), and there is probably a potential interactive regulatory mechanism between HK2 and Akt1 (p-Akt1) in cervical cancer cells. HK2 and Akt1 (p-Akt1) probably act as regulators of each other in cervical cancer cells and synergistically promote malignant growth and distant metastasis during the development of cervical cancer.

However, further research is still necessary to investigate the potential molecular mechanism through which HK2 upregulates Akt1 mRNA expression and stimulates p-Akt1 expression in cervical cancer cells. Numerous studies have demonstrated that the expression of MMP2 and MMP9 in cancer cells could be directly mediated by activation of the PI3K/AKT/mTOR pathway [39, 40]. Therefore, further research still needs to clarify whether the alteration of MMP2 and MMP9 expression in this study was directly regulated by FN1 stimulation or a result of Akt1 activation. Moreover, although the increased FN1 expression which followed with the activite of p-Akt1 had observed in this study, however, the opposite role of p-Akt1 on regulating FN1 expression also reported in breast cancer cells [41], the interaction between FN1 and p-Akt1 may be have a more complex manner. Finally, this study could not exclude the potential effect of Akt2 or Akt3 on regulating FN1 expression in HK2-modified cells, it still needs more experiments to conform in future study.

In conclusion, this study demonstrated that HK2 could activate Akt1 (p-Akt1) in cervical cancer cells, subsequently enhancing cell motility and tumor metastasis by inducing FN1, MMP2, and MMP9 expression. Furthermore, when the protein level of Akt1 (p-Akt1) was altered by using Akt1 recombinant plasmid or Akt1 inhibitor, the changing of HK2 expression was positive correlated with the Akt1 (p-Akt1) expression. Thereby, there is probably an interactive regulatory mechanism between HK2 and Akt1 (p-Akt1) during the malignant progression of cervical cancer (Fig. 4L).

## Supplementary Information

**Additional file 1: Fig. S1.** (A) The expression of HK2 between CESE (Cervical squamous cell carcinoma and endocervical adenocarcinoma) tissues and normal cervical tissues from GEPIA online database (http://gepia.cancer-pku.cn/). (B) The overall survival between HK2 high patients and HK2 low patients rom GEPIA online database (http://gepia.cancer-pku.cn/). (C) The mRNA expression of Akt1 was detected in HeLa-Vec, HeLa-HK2, SiHa-Vec and SiHa-HK2 cells by real-time quantitative PCR, and the quantitative analysis is shown; (D) The protein levels of Akt1 and p-Akt1 in HeLa-Vec cells were inhibited by the Akt1 inhibitor MK2206. The protein levels of Akt1, p-Akt1, HK2, FN1, MMP2 and MMP9 were detected by western blotting, and quantitative analysis is shown. (E) The protein levels of Akt1 and p-Akt1 in SiHa-Vec cells were inhibited by the Akt1 inhibitor MK2206. The protein levels of Akt1, p-Akt1, HK2, FN1, MMP2 and MMP9 were detected by western blotting, and quantitative analysis is shown. (F) The protein levels of Akt1, p-Akt1, HK2, FN1, MMP2 and MMP9 in HeLa-shCtr cells transiently transfected with an Akt1 recombinant plasmid were detected by western blotting, and quantitative analysis is shown. (G) The expression of MMP2 and MMP9 in cell medium supernatant was detected by the enzyme-linked immunosorbent assay (ELISA). (H) The protein levels of HK2, Akt1, p-Akt1, FN1, MMP2 and MMP9 in Ect-1/E6E7 cells transiently transfected with an HK2 recombinant plasmid were detected by western blotting, and quantitative analysis is shown. (I) An HK2 recombinant plasmid was transiently transfected into Ect-1/E6E7 cells, the migratory and invasive capacities were analyzed by the transwell assay. The data are shown as the mean ± SD of three independent experiments. * p < 0.05, ** p < 0.01 vs. control using one-way ANOVA. **Table S1.** The p-Akt1/Akt1 ratio in HK2-modified cells in this study.

## Data Availability

The transcriptomic dataset generated and analyzed during the current study are uploaded on https://www.ncbi.nlm.nih.gov/bioproject/PRJNA670405.

## Abbreviations

HK2: Hexokinases 2
FN1: Fibronectin
EMC: Extracellular matrix
SCC: Squamous cervical carcinoma
EMT: Epithelial-mesenchymal transition
MMP2: Matrix metalloproteinase-2
MMP9: Matrix metalloproteinase-9
IRS: Immunoreactive score
H&E: Hematoxylin and eosin
CESC: Cervical squamous cell carcinoma and endocervical adenocarcinoma
DMEM: Dulbecco’s modified Eagle’s medium
IHC: Immunohistochemistry
SFP: Specific pathogen-free
