# Supplementary material for: Hexokinases 2 promoted cell motility and distant metastasis by elevating fibronectin through Akt1/p-Akt1 in cervical cancer cells
Source: Cancer Cell Int. 2021 Nov 10;21:600. doi: 10.1186/s12935-021-02312-0 (PMC8579549; doi:10.1186/s12935-021-02312-0)
Supplement: Supplementary file 1 — Additional file 1: Fig. S1. (A) The expression of HK2 between CESE (Cervical squamous cell carcinoma and endocervical adenocarcinoma) tissues and normal cervical tissues from GEPIA online database (http://gepia.cancer-pku.cn/). (B) The overall survival between HK2 high patients and HK2 low patients rom GEPIA online database (http://gepia.cancer-pku.cn/). (C) The mRNA expression of Akt1 was detected in HeLa-Vec, HeLa-HK2, SiHa-Vec and SiHa-HK2 cells by real-time quantitative PCR, and the quantitative analysis is shown; (D) The protein levels of Akt1 and p-Akt1 in HeLa-Vec cells were inhibited by the Akt1 inhibitor MK2206. The protein levels of Akt1, p-Akt1, HK2, FN1, MMP2 and MMP9 were detected by western blotting, and quantitative analysis is shown. (E) The protein levels of Akt1 and p-Akt1 in SiHa-Vec cells were inhibited by the Akt1 inhibitor MK2206. The protein levels of Akt1, p-Akt1, HK2, FN1, MMP2 and MMP9 were detected by western blotting, and quantitative analysis is shown. (F) The protein levels of Akt1, p-Akt1, HK2, FN1, MMP2 and MMP9 in HeLa-shCtr cells transiently transfected with an Akt1 recombinant plasmid were detected by western blotting, and quantitative analysis is shown. (G) The expression of MMP2 and MMP9 in cell medium supernatant was detected by the enzyme-linked immunosorbent assay (ELISA). (H) The protein levels of HK2, Akt1, p-Akt1, FN1, MMP2 and MMP9 in Ect-1/E6E7 cells transiently transfected with an HK2 recombinant plasmid were detected by western blotting, and quantitative analysis is shown. (I) An HK2 recombinant plasmid was transiently transfected into Ect-1/E6E7 cells, the migratory and invasive capacities were analyzed by the transwell assay. The data are shown as the mean ± SD of three independent experiments. * p < 0.05, ** p < 0.01 vs. control using one-way ANOVA. Table S1. The p-Akt1/Akt1 ratio in HK2-modified cells in this study. [file 12935_2021_2312_MOESM1_ESM.doc]

**Additional Information**

**Figure S1:**

**
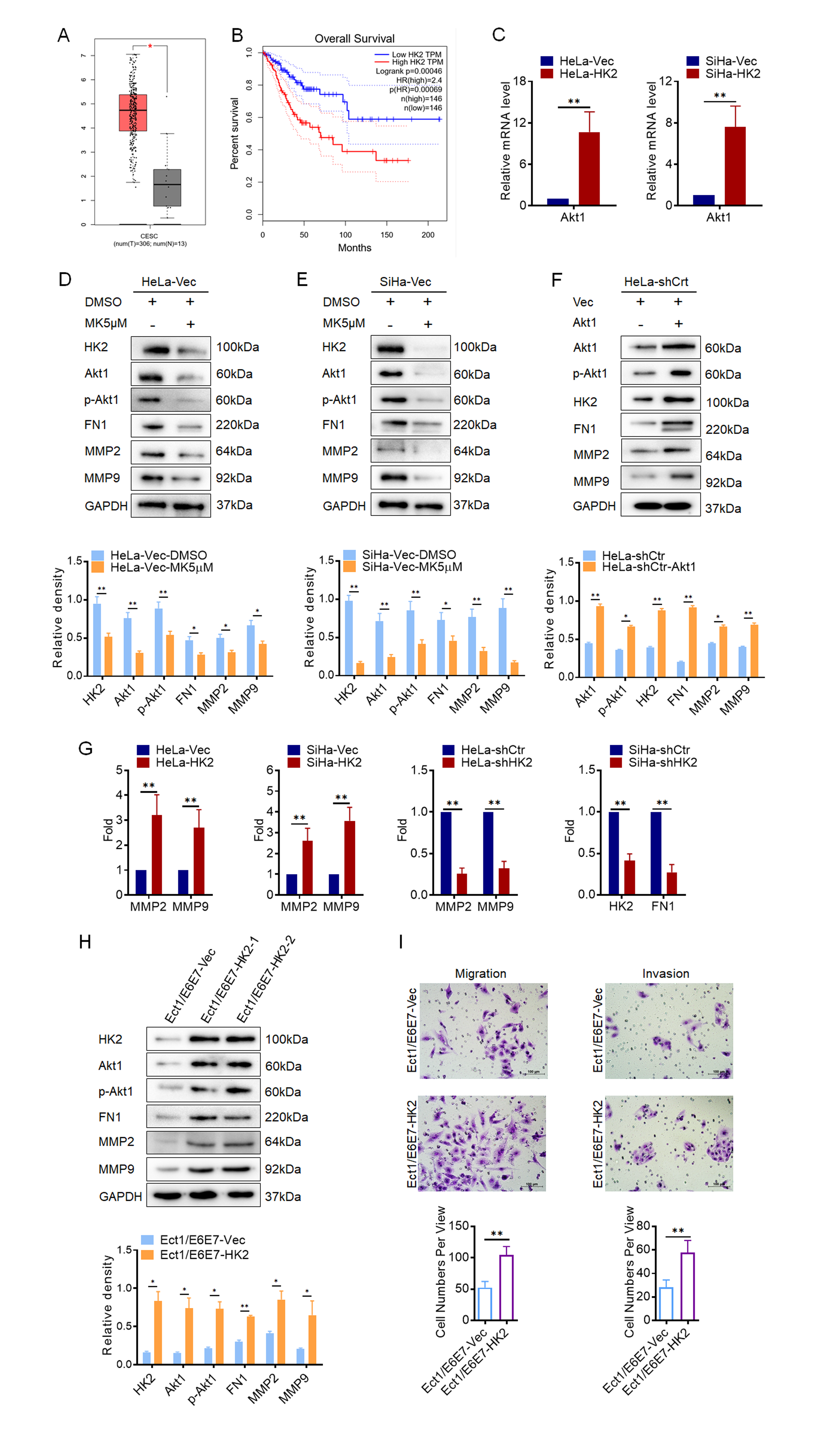
**

(A) The expression of HK2 between CESE (Cervical squamous cell carcinoma and endocervical adenocarcinoma) tissues and normal cervical tissues from GEPIA online database (http://gepia.cancer-pku.cn/). (B) The overall survival between HK2 high patients and HK2 low patients rom GEPIA online database (http://gepia.cancer-pku.cn/). (C) The mRNA expression of Akt1 was detected in HeLa-Vec, HeLa-HK2, SiHa-Vec and SiHa-HK2 cells by real-time quantitative PCR, and the quantitative analysis is shown; (D) The protein levels of Akt1 and p-Akt1 in HeLa-Vec cells were inhibited by the Akt1 inhibitor MK2206. The protein levels of Akt1, p-Akt1, HK2, FN1, MMP2 and MMP9 were detected by western blotting, and quantitative analysis is shown. (E) The protein levels of Akt1 and p-Akt1 in SiHa-Vec cells were inhibited by the Akt1 inhibitor MK2206. The protein levels of Akt1, p-Akt1, HK2, FN1, MMP2 and MMP9 were detected by western blotting, and quantitative analysis is shown. (F) The protein levels of Akt1, p-Akt1, HK2, FN1, MMP2 and MMP9 in HeLa-shCtr cells transiently transfected with an Akt1 recombinant plasmid were detected by western blotting, and quantitative analysis is shown. (G) The expression of MMP2 and MMP9 in cell medium supernatant was detected by the enzyme-linked immunosorbent assay (ELISA). (H) The protein levels of HK2, Akt1, p-Akt1, FN1, MMP2 and MMP9 in Ect-1/E6E7 cells transiently transfected with an HK2 recombinant plasmid were detected by western blotting, and quantitative analysis is shown. (I) An HK2 recombinant plasmid was transiently transfected into Ect-1/E6E7 cells, the migratory and invasive capacities were analyzed by the transwell assay. The data are shown as the mean±SD of three independent experiments. * p<0.05, ** p<0.01 vs. control using one-way ANOVA.

**Table S1. The p-Akt1/Akt1 ratio in HK2-modified cells in this study.**

| **Cell line** | **The p-Akt1/Akt1 ratio** |
| --- | --- |
| HeLa-Vec | 0.940± 0.067 |
| HeLa-HK2 | 1.511± 0.109 |
| SiHa-Vec | 0.912± 0.118 |
| SiHa-HK2 | 1.685±0.133 |
| HeLa-shCtr | 0.929± 0.014 |
| HeLa-shHK2 | 0.727±0.033 |
| SiHa-shCtr | 1.251±0.059 |
| SiHa-shHK2 | 0.915±0.039 |

HeLa-Vec *vs* HeLa-HK2: *p<0.05*,

SiHa-Vec *vs* SiHa-HK2: *p<0.05*,

HeLa-shCtr *vs* HeLa-shHK2: *p<0.05*,

SiHa-shCtr *vs* SiHa-shHK2: *p<0.05*.
